# Discussion on the New Code

**Published:** 1882-09

**Authors:** 


### Discussion on the New Code.

A considerable portion of our space in this nmmber of the Journal is devoted to an interesting discussion, which took place in The Buffalo Medical Club, on the Code of Ethics, recently adopted by the New York State Medical Society, a subject which has called forth in professional circles adverse as well as favorable criticism. We present both the affirmative and the negative arguments, with a view to offer both sides of this question, affording the reader the privilege of judging of the force of the respective arguments, and of drawing his own conclusions. The profession view this subject from widely different standpoints, according to individual bias. The prejudice of the past, if we may judge from the recent action of so able and respectable a body as the State Medical Society, is rapidly giving way to a more liberal professional sentiment. Whether this change is due to the force of public opinion, or to the approach of the various "pathies" towards a common therapeutical law, or to the greater tolerance of differences of opinion on vital principles, are suggestive questions. At any rate, it must be conceded that there is a deep significance in the action itself of the State Society. It betokens a widespread sentiment among our ablest medical men, that we should estimate professional skill and erudition and character in our consultations, rather than individual opinions of the action of curative agents, or the size of the doses which others may desire to administer. This idea is truly American and is the direct offshoot of our institutions.

The subject is of vast importance, and is justly creating discussion not only in this State, but throughout the land. If we may judge from the action of the American Medical Association, the general sentiment of the profession is adverse to the acceptance of this Code. The past teaches just such antagonisms to all great professional movements; and whether this initial step be the precursor of the breaking down of all barriers to professional intercourse, or to a general acceptance of uniformity of ideas in therapeutics, the future will decide. In the meantime the profession can well afford to look upon the new code with the spirit of forbearance at least, for if it subserves the great interests of humanity which it is the prerogative of medicine to foster and advance, the purpose of its enactment will be fully appreciated in the near future, and the State Society receive the endorsement of the profession. On the other hand, if its adoption was brought about through motives of *expediency* rather than of *principle*, the effort will prove one of those foolish attempts to reform the code without any reason therefor, and without any practical benefit to the profession or the public.

